# Supplementary material for: Association of sleep apnea with outcomes in peripheral artery disease: Insights from the PORTRAIT study
Source: PLoS One. 2021 Sep 10;16(9):e0256933. doi: 10.1371/journal.pone.0256933 (PMC8432750; doi:10.1371/journal.pone.0256933)
Supplement: S1 File — (DOCX) [file pone.0256933.s001.docx]

August 25, 2021,

Dear Editor,

We appreciate your review of our manuscript, “*Association of Sleep Apnea with Outcomes in Peripheral Artery Disease: Insights from the PORTRAIT Study*” for consideration for publication as a ‘Research Article’ in *PLOS ONE*.

We have addressed the additional concerns below:

1) We have confirmed that the following Data Availability Statement is acceptable: “Data contain potentially identifying information. The data are available upon request from the Yale Institutional Review Board. Institutional contact for data inquires: Gaelle Romain, PhD (Data Manager) Email Address: [gaelle.romain@yale.edu](mailto:gaelle.romain@yale.edu)”
2) Funding information for Boston Scientific and Terumo were removed from the updated funding disclosure statement for Dr. Smolderen because contracts are not active and were not sources of funding for the current study. None of Dr. Spertus’ conflicts of interests (Amgen, AstraZeneca, and copyrights to the PAG, SAQ, KCCQ) were sources of funding for the study.

Thank you again for your thoughtful review of our manuscript.

Sincerely,

**Kim G. Smolderen, PhD, FAHA**

Co-Director Vascular Medicine Outcomes (VAMOS) Program

Yale Medicine | Department of Internal Medicine | Section of Cardiovascular Medicine

789 Howard Avenue

New Haven, CT 06519

Email: [kim.smolderen@yale.edu](mailto:kim.smolderen@yale.edu)
