# Supplementary material for: Association of sleep apnea with outcomes in peripheral artery disease: Insights from the PORTRAIT study
Source: PLoS One. 2021 Sep 10;16(9):e0256933. doi: 10.1371/journal.pone.0256933 (PMC8432750; doi:10.1371/journal.pone.0256933)
Supplement: Attachment — Submitted filename: Response to Reviewers 7-26-2021.docx [file pone.0256933.s002.docx]

**ATTACHMENT 1: RESPONSE TO REVIEWERS**

**Responses to Reviewer #1 –**

1. *The use of questionnaires can be considered as a limitation of the study. Please discuss this point into a dedicated limitation section.*

We respectfully disagree with the reviewer on this comment. As research and clinical management of chronic disease populations shifts away from focusing only biomedical factors, methods have been developed to capture more patient-centered information. Validated patient reported outcomes measures (including the PAQ, EQ5D, GAD, PHQ etc used in this study) are high quality and rigorously tested tools developed using the classic test theory and also adopted by standards set by the FDA for the measurements of patient reported outcomes.^[1]^ These validated instruments are the gold standard for capturing patient reported outcomes and are a reliable and valid way to collect and study disease-specific health status and psychological constructs assessed in this study.

2. *The authors can also consider the impact of CPAP therapy on PAD outcomes. This would be really interesting as Ciccone et al. [Int J Cardiol. 2012 Jul 26;158(3):383-6] previously demonstrated the reversibility of endothelial function after CPAP therapy.*

We agree that adding the implications of treatment with CPAP to the discussion would add to the manuscript. We have added the following commend and citation to the Discussion section [page 16, line 274]:

*“Regardless of whether sleep apnea can be considered an independent risk factor of adverse outcomes for PAD or whether it is a marker of underlying atherosclerotic risk, it is important to emphasize that treatment of sleep apnea (weight loss, behavioral therapy including alcohol cessation, CPAP and surgical correction) [56] can potentially lead to improved outcomes, including patients’ health status and psychological outcomes such as depressive symptoms in patient with hypertension, atrial fibrillation and heart failure [40-42]. In addition, CPAP treatment has also been shown to improve endothelial function after initiation [57].”*

3. *The duration of sleep apnea may impact on the severity of the PAD. Please discuss such a point in relation to the paper from Ciccone MM et al. Respir Med. 2012 May;106(5):740-6.*

We agree that the duration of sleep apnea may have an impact on the severity of PAD. We have added this as a limitation in the Discussion section [page 17, line 313] as our study is unable to associate the duration of sleep apnea with features of the patient’s PAD as follows:

*“We were unable to assess whether the duration of having comorbid sleep apnea was associated with the severity of PAD, as increased duration of disease has been linked with increasing intima thickness in carotid stenosis [76].”*

**Responses to Reviewer #2 –**

1. *Methods page 5 line 18: please provide the reference about Rutherford Classification*.

The citation for the Rutherford Classification (Rutherford, 1997) has been added.

2. *“Could you please provide a comparison between patients with different Rutherford classes (3,4,5 and 6) with and without sleep apnea? PAD includes very different patterns of disease which can not be put together in the same analysis.”*

We agree that there is variation in PAD by Rutherford Classification, however we are unable to provide the analysis requested as the PORTRAIT Registry only included patients with symptomatic claudication (Rutherford Class 1, 2, and 3), and Rutherford 4-6 was an exclusion criterion. In our results, we have included a comparison between those with and without sleep apnea by symptom severity (mild, moderate, and severe claudication symptoms), and we found this association to not be statistically significant (p = 0.55). The Rutherford Classification labels corresponding to those symptoms (Class 1, 2, and 3 respectively) have been added to Table 1 for clarity [page 9-10 with changes on page 10 and 11].

3. *“The authors should also stratify the results between patients who were revascularized and those who were not in order to make the results more homogeneous.”*

We stratified the health status outcomes by management strategy (revascularization versus conservative management without revascularization) and did not find a statistically significant difference. This information was added to the manuscript as follows:

Methods [page 8, line 166]

*“We also tested the interaction between sleep apnea and two other variables: (1) CPAP use (yes versus no) and (2) initial management strategy (revascularization versus conservative management without revascularization) to the unadjusted models to verify whether differences in health status outcomes existed for those who were treated with CPAP or revascularization versus those who were not.”*

Results [page 13, line 226]

*“There were no differences in health status outcomes between sleep apnea patients using CPAP versus those with sleep apnea without CPAP treatment or between sleep apnea patients treated with revascularization versus those with sleep apnea who were managed conservatively (without revascularization) in the unadjusted model so no further modeling including these interaction terms was performed.”*

1. US Department of Health and Human Services FDA Center for Drug Evaluation and Research laurie. burke@ fda. hhs. gov, US Department of Health and Human Services FDA Center for Biologics Evaluation and Research toni. stifano@ fda. hhs. gov, & US Department of Health and Human Services FDA Center for Devices and Radiological Health SXD@ cdrh. fda. gov. (2006). Guidance for industry: patient-reported outcome measures: use in medical product development to support labeling claims: draft guidance. *Health and Quality of Life Outcomes*, *4*, 1-20.
